# Moonlighting of mitotic regulators in cilium disassembly

**DOI:** 10.1007/s00018-021-03827-5

**Published:** 2021-04-15

**Authors:** Cenna Doornbos, Ronald Roepman

**Affiliations:** 1 Department of Human Genetics, Radboud University Medical Center, Nijmegen, The Netherlands; 2 Radboud Institute for Molecular Life Sciences, Radboud University Medical Center, Nijmegen, The Netherlands

**Keywords:** Cilia, Cilium resorption, WNT, Cell cycle regulators, Centrioles, Tumour development

## Abstract

Correct timing of cellular processes is essential during embryological development and to maintain the balance between healthy proliferation and tumour formation. Assembly and disassembly of the primary cilium, the cell’s sensory signalling organelle, are linked to cell cycle timing in the same manner as spindle pole assembly and chromosome segregation. Mitotic processes, ciliary assembly, and ciliary disassembly depend on the centrioles as microtubule-organizing centres (MTOC) to regulate polymerizing and depolymerizing microtubules. Subsequently, other functional protein modules are gathered to potentiate specific protein–protein interactions. In this review, we show that a significant subset of key mitotic regulator proteins is moonlighting at the cilium, among which PLK1, AURKA, CDC20, and their regulators. Although ciliary assembly defects are linked to a variety of ciliopathies, ciliary disassembly defects are more often linked to brain development and tumour formation. Acquiring a better understanding of the overlap in regulators of ciliary disassembly and mitosis is essential in finding therapeutic targets for the different diseases and types of tumours associated with these regulators.

**Supplementary Information:**

The online version contains supplementary material available at 10.1007/s00018-021-03827-5.

## Introduction

Primary cilia are small organelles protruding from the plasma membrane. Their immobility distinguishes them from their motile counterparts, that have a clear function in extracellular fluid propulsion. Primary cilia are conserved across a variety of species and are present on almost every mammalian cell. These cilia have evolved into cellular signalling hubs by harbouring components of critical cell signalling pathways, such as the ‘Wingless and Int-1’ (WNT) signalling pathway [1, 2], ‘Sonic hedgehog’ (SHH) [3, 4], and autophagy [5, 6]. The exact ciliary signalling functions of the primary cilia vary widely and depend on the developmental stage and cell type. Due to their near-ubiquitous prevalence, dysfunction of the cilia can disturb the formation and functioning of a variety of organs, and therefore, is linked to a wide, overlapping spectrum of hereditary disorders denominated “ciliopathies” [7].

**Fig. 1:**
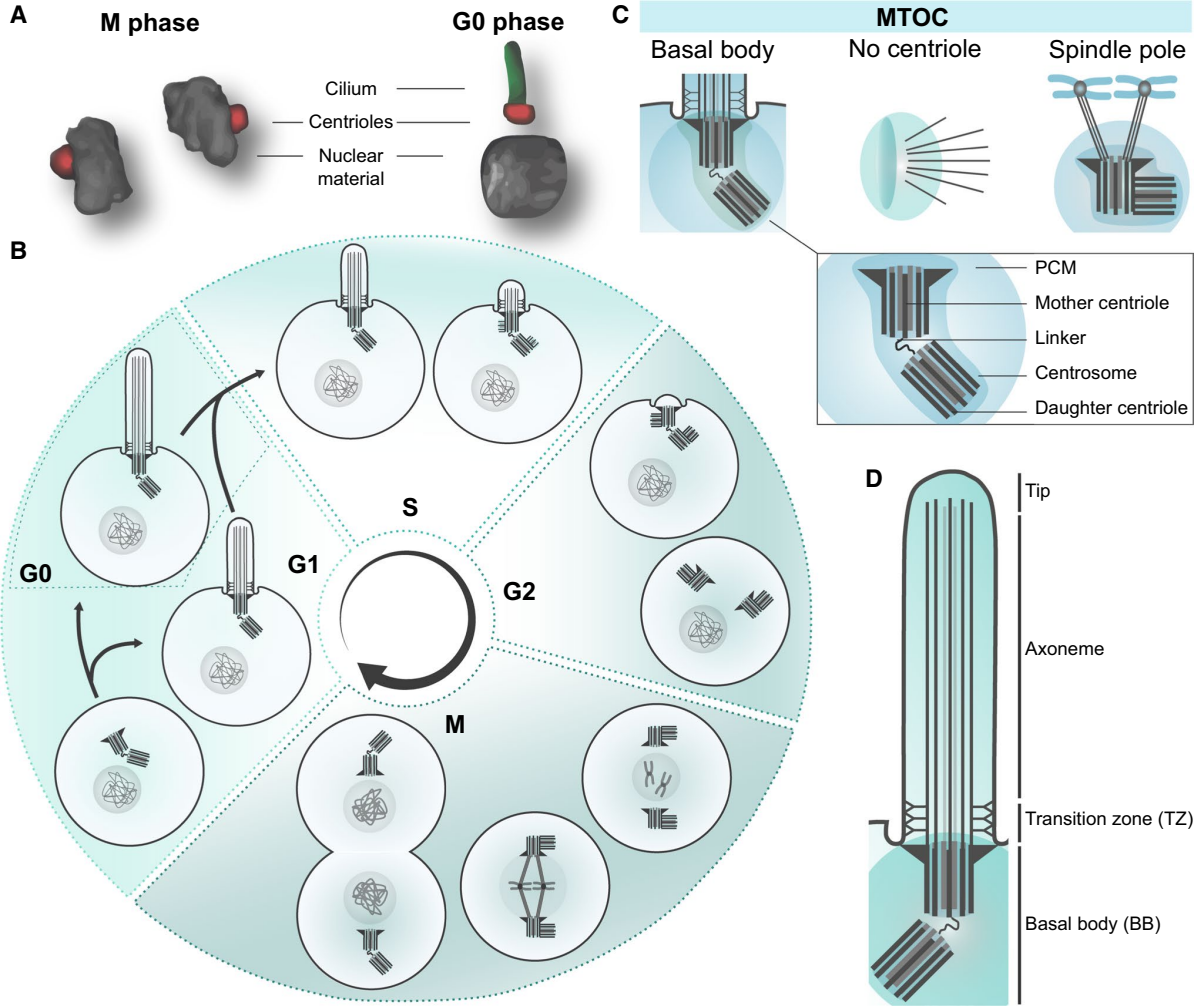
Cilia and ciliation cycle. **a** Graphic representation of the centrioles. During mitosis (M phase) the centrioles form the spindle poles to separate the nuclear material. In quiescence (G0 phase) the centrioles (red) are positioned at the base of the cilium (green). **b** The cilia assemble every cell cycle in G0/G1 phase and disassemble during S/G2 phase, during which the centrioles are positioned at the ciliary base. After detachment from the plasma membrane, the linker between the mother and daughter centriole dissolves, allowing the two centrioles to move towards the nucleus to form the spindle poles. Each mother centriole forms a new daughter centriole during the next cycle. **c** The centrosomes, displaying two structurally different centrioles surrounded by the pericentriolar material (PCM), act as the main MTOC both during spindle pole formation and ciliogenesis. Nonetheless, an MTOC can also arise without a centrosome [174]. The spindle pole and basal body refer to the centrosome as an MTOC with the pericentriolar material, but either contain proteins specific to the cell cycle, or ciliogenesis. The centrosomes gather different complexes for these specific functions, enriching the PCM for different sets of proteins, and making it more likely for interacting proteins to bind at the right phase of the cell cycle. **d** Schematic representation of the cilia and the ciliary regions that can be distinguished

Next to its general role as a cellular signalling hub, a more specific function of cilia in cell cycle regulation has become increasingly pronounced. Several studies suggest a link between cilia, tumour formation, and in some cases, mosaic variegated aneuploidy (MVA) syndrome [8–11]. This link is best explained by the important double role of the centrosomal centrioles in both segregation of the sister chromatids during cell division and in ciliogenesis during the G0/G1 phase of the cells cycle (Fig. 1a). As these roles of the centrioles are mutually exclusive, it requires the assembly and disassembly of the cilia each round of the cell cycle (Fig. 1b). The terms centriole, centrosome, microtubule-organizing centre (MTOC), spindle pole and basal body are often used intertwined, but they do not always refer to the same structures (Fig. 1c). After facilitating segregation of the nuclear material during M phase, the centrioles migrate towards the plasma membrane. Here, the mother centriole docks to the plasma membrane using the distal appendages and drive the accumulation of ciliogenesis-specific proteins towards the pericentriolar matrix (PCM) to form the basal body, from which the cilium extends [12, 13]. When the cilia are present at the cell membrane, the centrioles cannot migrate back to the nucleus to facilitate mitosis, therefore, cell cycle re-entry requires the release of the centrioles from the membrane to enable reconstruction of the mitotic spindle poles.

The primary cilium is a highly organized organelle (Fig. 1d) [14], and ciliary assembly and disassembly are complex, well-timed mechanisms that regulate the formation and breakdown of each ciliary component. Even though the basal body forms the root of the cilium, it also functions as a protein recruitment centre. Several mechanisms cooperate to deliver proteins to the ciliary base in a tightly regulated fashion. The ciliary membrane has a different composition than the plasma membrane and this is regulated at the ciliary pocket and transition zone (TZ) [15, 16]. The membrane of the cilium is enriched for signalling molecules and cilium-specific membrane regulators. Other proteins that are not membrane-associated can be recruited from the cytoplasm to the centriolar satellites [17, 18]. To assure correct protein trafficking, a gating module is localized at the TZ just above the basal bodies [19, 20]. Active transport along the microtubules (MTs) that make up the ciliary axoneme is regulated by two sets of ‘Intraflagellar transport’ (IFT) proteins: IFTA for retrograde transport to the ciliary base using dynein motor proteins and IFTB for anterograde transport to the tip of the cilia using kinesin motor proteins [21]. This strict regulation is also seen in ciliary maintenance during G0–G1 phase, by a constant balance between ciliary assembly and disassembly [22].

## Ciliary disassembly

**Fig. 2:**
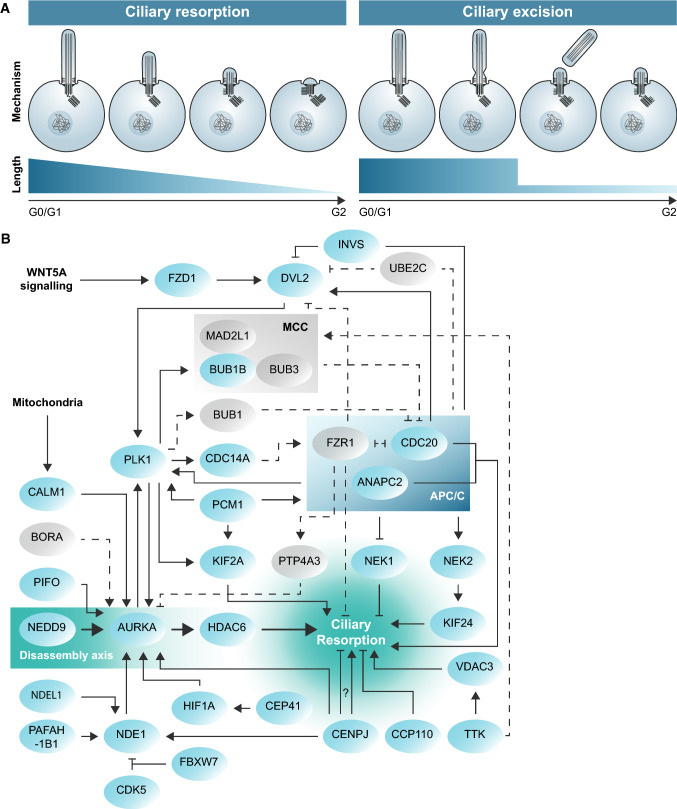
Ciliary disassembly mechanism. **a** A schematic representation of ciliary resorption versus ciliary excision. **b** The core axis of ciliary disassembly is regulated through the NEDD9/AURKA/HDAC6 pathway that drives ciliary resorption. This axis can be stimulated by the mitochondria, extracellular WNT signalling and cell cycle regulators. The criteria for a protein to be considered a ciliary resorption protein, and to be included in this schematic overview, the protein has to influence ciliary length during disassembly, but is not involved in ciliary excision or budding. All of these proteins with a confirmed role in ciliary resorption are shown in blue. In addition to these, proteins shown in grey and with dotted lines indicate known interactors of these resorption proteins, but which have not yet been investigated in the context of ciliary resorption specifically. PLK1 both stimulates and inhibits ciliary resorption by phosphorylation of a wide variety of targets. The APC/C stimulates ciliary resorption when activated by CDC20. For CENPJ, the exact role in ciliary resorption remains elusive

Cilia can disassemble by either cilium resorption or cilium excision (Fig. 2a). During resorption, the primary cilium is broken down gradually at variating velocities and all components are resorbed by the cell [23–25]. On the other hand, during excision the membrane of the cilium is pinched to shed part of the cilium [25]. The latter is also called whole cilium shedding and is not to be confused with ciliary vesicle shedding, in which small membranous vesicles are released extracellularly from the cilium [26–28]. Ciliary excision was previously reported in *Chlamydomonas reinhardtii* in which stress allowed to shear off the whole cilium at the TZ [29, 30]. This mechanism can be induced through an acidity shock or by Dibucaine treatment, leaving a cilium-enriched fraction after centrifugation of the growth medium [30–32]. More recent reports have shown that this mechanism of cilium excision is also conserved in mammalian cells [25]. Although the role of primary cilium excision in mammalian cells requires further exploration, the significance of resorption as a mechanism of ciliary disassembly is well established and it has been shown that cilium resorption is essential for cell survival in cycling cells [33].

### The core axis in ciliary resorption is stimulated through WNT

For a protein to be considered a ciliary resorption protein, it has to been proven that it influences ciliary length during ciliary disassembly, while it is not involved in ciliary excision or budding. The core players in ciliary resorption are represented by the NEDD9/AURKA/HDAC6 axis, located at the base of the cilia (Fig. 2b). HDAC6 destabilizes ciliary MTs through deacetylation [34, 35]. HDAC6 localizes the MTOC organizing protein AURKA to the basal body, where it is stabilized by NEDD9 (previously known as HEF1) [23, 36]. Since most proteins discussed here have multiple names, all proteins names, alternative names and their corresponding IDs are summarized in Electronic Supplementary Material—Table 1. NEDD9 stabilization prevents ubiquitin–proteasome system (UPS)-mediated degradation of AURKA [37]. In turn, AURKA promotes HDAC6-mediated axoneme destabilization, functioning as a feedforward loop in ciliary disassembly.

The NEDD9/AURKA/HDAC6 axis is promoted through multiple pathways, among which mitochondrial stimulation through CALM1 [38, 39]. Another example is autophagy, since serum-starvation is often used in cell culture to induce cell cycle arrest and stimulate cilium formation [40]. On the other hand, serum resupplementation is associated with MTOR activation, ciliary disassembly and cell cycle re-entry. AURKA activation is also mediated by PCM1, which recruits PLK1 to the basal body during ciliary disassembly [41]. Furthermore, PIFO-dependent AURKA activation promotes ciliary disassembly and is linked to left–right symmetry patterning [42, 43]. Last, the best-described pathway to stimulate ciliary resorption is WNT signalling. WNT signalling promotes cell cycle division and is essential in embryonic development, but also stimulates the NEDD9/AURKA/HDAC6 axis [44]. Extracellular WNT5A activates FZD1 on the cell membrane to promote DVL2 signalling [45]. In turn, DVL2 promotes PLK1-dependent phosphorylation of AURKA, resulting in more potent deacetylation by HDAC6. Furthermore, PLK1 enhances the MT depolymerisation through kinesin motor protein KIF2A in ciliary disassembly [46]. Thus far, one potentially pathogenic KIF2A mutation has been found in patients with microcephaly [47].

There are several WNT5A mutations that are linked to Robinow syndrome. Robinow has previously been indicated as a possible ciliopathy due to the overlap in symptoms compared to skeletal ciliopathies [48]. It would, therefore, be interesting to determine what the molecular influence is of these WNT5A mutations on ciliary disassembly and function. Another hypothesized ciliopathy causing mutation is the premature stop mutation 282A > T in *HDAC6, since it* causes chondrodysplasia, brachydactyly, hydrocephaly and microphthalmia (Table 1) [49]. Furthermore, as part of the core disassembly axis, a tight regulation of NEDD9 and AURKA is essential. For NEDD9, there are no known pathogenic mutations, however, high protein levels are associated with breast cancer [37]. High protein levels of ARUKA are associated with several types of tumours and poor survival rates [50, 51]. Overall, AURKA is considered the key link between ciliary disassembly and tumour development, which has recently been reviewed in depth [52].

**Table 1:** Ciliary resorption regulators and related diseases

Name	OMIM disease	Genetic variants (pathogenic)	Resorption regulator
ANAPC2	–	3 (0)	↑
AURKA	CRC (MIM 114500)	3 (2)	↑
BUB1B	MVA (MIM 257300)	234 (20)	↓
	PCS (176430)		
	CRC (MIM 114500)		
CALM1	LQT (MIM 616247)	45 (14)	↑
	CPVT (MIM 614916)		
CCP110	–	10 (0)	↓
CDC14A	DFNB (MIM 608653)	37 (11)	↓
CDC20	–	2 (0)	↑
CDK5	LIS (MIM 616342)	9 (1)	↓
CENPJ	MCPH (MIM 608393)	149 (44)	↑↓?
	SCKL (MIM 613676)		
CEP41	JBTS (MIM 614464)	182 (15)	↑
DVL2	–	5 (0)	↑
FBXW7	–	31 (14)	↓
FZD1	–	2 (0)	↑
HDAC6	Ciliopathy^1^ (MIM 300863)	33 (1)	↑
HIF1A	–	14 (0)	↑
INVS	NPHP (MIM 602088)	239 (45)	↓
KIF24	–	11 (0)	↑
KIF2A	CDCBM (MIM 615411)	75 (7)	↑
NDE1	MHAC (MIM 605013)	97 (17)	↑
	LIS (MIM 614019)		
NDEL1	–	0 (0)	↑
NEDD9	–	3 (0)	↑
NEK1	SRTD/SRPS (MIM263520)	202 (47)	↓
	ALS (MIM 617892)		
NEK2	RP (MIM 615565)	11 (1)	↑
PAFAH1B1	LIS (MIM 607432)	257 (124)	↑
PCM1	–	20 (0)	↑
PIFO	–	0 (0)	↑
PLK1	–	1 (0)	↑↓
TTK	–	6 (1)	↑
VDAC3	–	0 (0)	↑
WNT5A	DRS (MIM 180700)	113 (11)	↑

An overview of all validated ciliary resorption proteins, as indicated in blue in Fig. 2b, and weather these positively (↑) or negatively (↓) regulate ciliary resorption. For a protein to be included here as a ciliary resorption regulator, it has to meet the criteria that it influence ciliary length during ciliary disassembly, while it is not involved in ciliary excision or budding. The genetic variants indicate the total number of ClinVar genetic variants affecting only this gene (excluding multigene insertions, deletions and copy number variants). Between () is the number of these variants that are indicated as ‘Likely pathogenic’, ‘Pathogenic’, ‘Risk factor’ or ‘Conflicting interpretations’. For CENPJ, there are conflicting reports whether it is a resorption promotor or repressor.

*ALS* amyotrophic lateral sclerosis, *CDCBM* cortical dysplasia, complex, with other brain malformations, *CPVT* ventricular tachycardia, catecholaminergic polymorphic, *CRC* colorectal cancer, *DFNB* deafness, with or without immotile sperm, *DRS* robinow, *JBTS* Joubert syndrome, *LIS* lissencephaly, *LQT* long QT syndrome, *MCPH* microcephaly, *MHAC* microhydranencephaly, *MVA* mosaic variegated aneuploidy syndrome, *NPHP* nephronophthisis, *PCS* premature chromatid separation, *RP* retinitis pigmentosa, *SCKL* Seckel syndrome, *SRPS* short-rib polydactyly syndrome, *SRTD* short-rib thoracic dysplasia

^1^The referred mutation in HDAC6 might cause a ciliopathy, since patients present with common ciliopathy symptoms; chondrodysplasia with platyspondyly, distinctive brachydactyly, hydrocephaly, and microphthalmia.

### The cell cycle complex APC/C plays a key role in ciliary resorption

Next to its role in anaphase, the E3 ubiquitin ligase APC/C is also involved in ciliary disassembly. Its complex member ANAPC2 and APC/C co-activator CDC20 promote ciliary disassembly, potentially by targeting ciliary components for UPS-mediated degradation [53]. The mitotic checkpoint complex (MCC) cell cycle regulator, consisting of BUB1B, BUB3 and MAD2L1, is thought to competitively bind CDC20, to prevent APC/C-CDC20 complex formation during G0/G1 phase [54]. BUB1B has been shown to function as a negative regulator of ciliary disassembly [54]. Next to CDC20, the APC/C can also be activated through FZR1 (previously known as CDH1), to target a different set of proteins. Furthermore, CDC20 knockdown and FZR1 overexpression resulted in reduced DVL2 levels [54] and ANAPC2 is required for cilia polarity through DVL2-WNT signalling [55]. Together these data suggest that APC/C-CDC20 is a disassembly promoter, while APC/C-FZR1 might inhibit ciliary disassembly.

The role of PLK1 in this process remains unclear. PLK1 depletion results in decreased CDC14A-dependent dephosphorylation of FZR1 [56] and CDC14A is a positive regulator of ciliogenesis [57]. Furthermore, PLK1 activity is promoted by the APC/C, which both support the idea that FZR1 might be a ciliary resorption inhibitor. On the other hand, during M phase, APC/C-CDC20 targets PLK1 and BUB1B for proteasomal degradation upon correct kinetochore attachment, to allow mitotic exit [58, 59]. Mutations in BUB1B are associated with MVA, colorectal cancer (CRC), and premature chromatid separation (PCS), but there are no known pathogenic patient mutations for PLK1 [60, 61]. Together, these data indicate that the exact mechanism of PLK1 and APC/C regulation is dependent on the specific spatiotemporal conditions and that their role might differ if the cell is in mitosis or performing ciliary disassembly.

The APC/C regulates its function in ciliary disassembly through multiple targets. One group is the family of ‘never in mitosis gene A’ ‘(NIMA) related kinase’ (NEK) family of kinases, that are thought to function as cell cycle and checkpoint control proteins [62]. NEK1 promotes centrosome stability during ciliogenesis and is associated with polycystic kidney disease (PKD) [63], and skeletal ciliopathies [64]. NEK1 is linked to ciliary tubulin organization through CEP104, while APC/C-mediated degradation of NEK1 is required for ciliary resorption [53, 65, 66]. In addition, NEK2-mediated activation of KIF24 is required for ciliary disassembly [67] and incorrect functioning of NEK2 is related to the ciliopathy retinitis pigmentosa (RP) [68]. Overall, the family of NEK proteins is associated with different types of tumours [62].

Next to the NEK proteins, the APC/C can influence ciliary resorption through AURKA activation or degradation. In turn, this influences HDAC6-regulated ciliary disassembly through INVS, which marks the ciliary INVS/NPHP3 compartment, located just above the TZ in the cilium. Mutations in *INVS* cause the ciliopathy Nephronophthisis (NPHP) type 2 [69, 70]. It binds ANAPC2 to promote DVL2 degradation and, as a consequence, inhibits AURKA phosphorylation by PLK1 [71]. In mature human retinal pigment epithelium (RPE) cells it has been shown that this mechanism is used in fully differentiated cells to inhibit ciliary disassembly by recruiting INVS to the base of the cilium [72]. Interestingly, INVS also binds directly to yet another NEK protein family member, NEK8, recruiting it to the ciliary INVS/NPHP3 compartment [73]. This recruitment requires hydroxylation of INVS and ANKS6 by HIF1AN [74], and mutations in *NEK8* underly the renal ciliopathy NPHP type 9. NEK8 is important for cell cycle regulation through the Hippo signalling pathway, and in turn, the Hippo and WNT pathways closely interact to regulate gene transcription [75, 76]. Nonetheless, it has not yet been investigated if NEK8 is also a target of direct APC/C-mediated degradation, like NEK1. In conclusion, the APC/C has many interactors to regulate the cell cycle and ciliary resorption, but whether it is a ciliary resorption promotor or repressor might depend on the co-activator that is bound.

### Centrosomal proteins are involved in ciliogenesis, ciliary resorption and ciliary excision

The centrosomal core proteins travel along with the centrioles from the spindle poles in M phase to the ciliary base in G1/S phase. It has been shown that the depletion of centrosome-associated proteins, among which PCM1, tubulin, and NEK2, results in a decrease in ciliogenesis [77]. In this process the loss of centrosome integrity results in G1 to S phase cell cycle arrest through MAPK14 (p38α), TP53 and CDKN1A (p21) cell cycle regulators [77], possibly by disruption of CEP131, PCM1 and CEP290 localization in the centriolar satellites [78]. PCM1 seems to be essential for both ciliogenesis and ciliary disassembly, most likely through its role in the recruitment of proteins to the basal body and centriolar satellites. On the other hand, centrosomal protein CEP41 blocks HIF1A UPS-mediated degradation and in turn, HIF1A stimulates AURKA phosphorylation, which promotes ciliary resorption [79]. Interestingly, HIF1AN is, next to its role in NEK8 localization, a direct inhibitor of some HIF1A interactions under normoxia conditions [80], making it an interesting target to study with regards to ciliary resorption and cellular oxygen-sensing. Another centrosomal protein, CENPJ (also known as CPAP or SAS4) has opposing roles during ciliary disassembly and mitosis, which seems to be a characteristic for disassembly-related proteins.

CENPJ has been indicated to promote ciliary resorption and is essential for maintenance of the neural progenitor pool by forming a scaffold for NDE1, AURKA and OFD1 [81]. In contrast, others have reported that it promotes centriole elongation and is required for centriole duplication in late mitosis [82, 83]. This led to the opposing finding that CENPJ is a promoter of ciliogenesis and its expression decreased upon the induction of ciliary disassembly, which was not seen for disassembly proteins AURKA, PLK1 and NEK2 [84]. CENPJ is associated with Seckel syndrome (SCKL) and Microcephaly (MCPH) [85, 86]. The precise function of CENPJ in ciliary resorption might be dependent on its interacting proteins at the centrosomes.

CCP110 (also known as CEP110) has been shown to supress cilium assembly in conjunction with CEP97 by capping the mother centriole [87], and by association with a complex of other centrosomal proteins, among which CEP290 (NPHP6), RAB8A, CEP104, and CENPJ [88–92]. In turn, CEP290, NPHP4, RPGRIP1L (NPHP8), TMEM107 and TMEM216 have been indicated in ciliary excision [93]. Mutations in CEP290 cause a variety of ciliopathies, including ‘Joubert syndrome’ (JBTS) [94], ‘Leber congenital amaurosis’ (LCA) [95] and ‘Meckel syndrome’ (MKS) [96]. Furthermore, RPGRIP1L [97, 98], TMEM107 [99, 100] and TMEM216 [101, 102] malfunctioning are also associated to JBTS and MKS among others, while mutations in NPHP4 [103, 104] are associated with NPHP and ‘Senior-Loken syndrome’ (SLS) ciliopathies. Overall, centrosomal proteins seem to have a role in ciliogenesis, ciliary resorption and ciliary excision and are associated with a range of ciliopathies, while other ciliary resorption proteins are more often associated with microcephaly and tumour formation.

In summary, ciliary resorption depends upon a complex interplay of proteins including the APC/C and WNT signalling. Many of the proteins involved in ciliary resorption also play a role in cell division and disruption of these mechanisms can lead to microcephaly and a variety of cancers. The centrosomal proteins at the base of the cilium also seem to affect ciliary resorption, but ciliary excision and ciliogenesis as well. Therefore, the mechanisms involved in cilium resorption might be different from the ones involved in ciliary excision, which is underpinned by the fact that ciliary excision proteins are associated with a variety of ciliopathies, even though ciliary resorption proteins are more often associated with tumour formation and microcephaly. The latter might be explained by the double role of many ciliary resorption proteins in mitosis. Nonetheless, we do not fully understand how these proteins specifically disturb brain formation during embryology rather than affecting all mitotic process.

## Mitotic structures: kinetochores and spindle poles

**Fig. 3:**
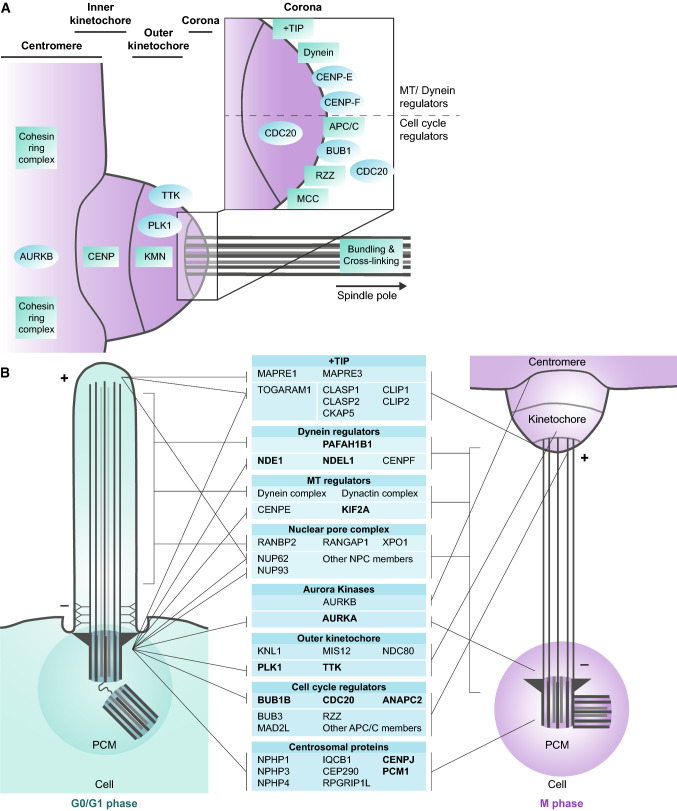
Conservation between the cilia, kinetochores and spindle poles. **a** Schematic representation of the kinetochore, and the key proteins present in each part of this structure. Individual proteins are indicated as circles (blue), protein complexes as squares (green). The MTs are docked onto the outer kinetochore. The corona contains many different proteins and protein complexes (inset), which either affect MT and dynein organization or cell cycle regulation. The MTs are bundled and crosslinked to withstand the high mechanical forces between the kinetochores and spindle poles prior to and upon segregation of the sister chromatids. **b** Many proteins and protein complexes are conserved between the cilia, kinetochores and spindle poles. Proteins that have been confirmed to play a role in ciliary resorption are marked (bold). The proteins are sorted per module and their organization in the two structures seems to be dependent on the MT organization, from minus at the bottom to plus at the top

After ciliary disassembly, the centrioles migrate back to the nucleus, where they behave again as the main MTOC by nucleating an assembly of MT-polymerizing proteins that form the spindle poles. The plus-ends of the MTs are bound to the kinetochore with the help of the fibrous corona. The kinetochore consists of three layers: the corona, the outer kinetochore and the inner kinetochore (Fig. 3a). Kinetochore function and architecture have been reviewed in detail [105–109]. In summary, the corona is required for the correct positioning of the chromosomes towards the equatorial plane by binding of the MTs that extend from the MTOC. Since it is more likely to bind an MT at the side, rather than at the end, the corona provides a lateral to end-on conversion with the help of motor proteins [110]. CENPF recruits the NDE1/NDEL1/PAFAH1B1 (also known as LIS1) dynein motor complex to the kinetochore [111]. Both the minus-end-directed motor protein dynein and the plus-end-directed CENPE protein are required for correct localization of the kinetochores at the MT plus-ends [112, 113].

The kinetochores are aligned on the equatorial plane in bi-orientation to assure that each sister chromatid is attached to the opposite spindle pole. BUB1 recruits the KNTC1 (also known as ROD)-ZWILCH-ZW10 (RZZ) complex and BUB1B proteins to the kinetochores for the regulation of MT embedding and chromosome segregation [114]. Chromosome segregation and the onset of anaphase are strictly inhibited by the spindle poles until each kinetochore is attached to MTs [115]. Upon correct attachment of all kinetochores, CDC20 activates the APC/C to polyubiquitinate PTTG1 (Securin), marking it for UPS-mediated degradation. In turn, PTTG1 can no longer inhibit ESPL1 (Separin), which cleaves the Cohesin ring complex that keeps the two sister chromatids together. This precise regulation is essential, since chromosome missegregation is linked to MVA and tumour formation [60, 61]. When the kinetochore is positioned end-on, the KNL1/MIS12/NDC80 (KMN)-complex embeds the plus-ends of the MTs into the outer kinetochore [116]. During mitosis, the kinetochore is attached to the centrosomes with the help of the inner kinetochore centromere protein (CENP) family, of which the members are rich in DNA-binding motifs.

The link between the MTOC and the kinetochores is strengthened by the formation of kinetochore fibres (k-fibres). These fibres are formed by bundling and cross-linking of the MTs, and are required to withstand the high mechanical forces involved in chromosome segregation. Incorrect cross-linking of the MTs to form the k-fibres and mutations in depolymerising MT-tracking protein CENPF have been linked to microcephaly [117–119]. Intriguingly, this phenotype is seen in a variety of ciliopathies, among which ‘Mental retardation, truncal obesity, retinal dystrophy, and micropenis’ (MORM), suggesting a functional overlap of the proteins involved in k-fibre formation [120].

At the end of M phase, the kinetochores are dissociated, the MTs depolymerised, and the centrioles are released [121–123]. It is interesting to see that upon the formation of a new cilium in the next G1 phase, not only the centrioles, but also many of these kinetochore proteins play a role in ciliary assembly, disassembly, or functioning.

## Conservation between cell cycle and ciliary disassembly proteins

We have briefly touched upon the double role of some cell cycle regulators in ciliary disassembly and vice versa. If we look at the structural conservation between the cilia and kinetochores, we can see that the further you go towards the outside of the kinetochores, the more proteins you will find that play a role in ciliary disassembly (Fig. 3b).

### Outer kinetochore proteins, but not inner kinetochore proteins, play a role in ciliary disassembly

Starting at the inner part, none of the inner kinetochore proteins have been detected in cilia. This is best explained by the role of the inner kinetochore proteins in binding of the kinetochore to the DNA. Since this is not required in cilia formation, the CENP family of DNA-binding proteins are not found in cilia. However, there are three CENP family members, CENPJ, CENPF, and CENPE that do have a function in ciliogenesis or ciliary disassembly. Nonetheless, these three proteins do not have DNA-binding motifs and are not localized at the inner kinetochore, but at the spindle poles or corona instead.

Furthermore, the outer kinetochore proteins play an important role in ciliary disassembly and cell division. PLK1 is one of the proteins that is rooted deeply in both processes. In addition, cell cycle protein TTK (also known as MPS1) also plays a role in ciliary disassembly. During M phase, TTK is required for the localization of CENPE to the kinetochores [124, 125]. Here, it regulates APC/C-CDC20 activity through MAD2L1 of the MCC. After cell division, TTK moves to the base of the cilia, where it is required for recruitment of mitochondrial channel protein VDAC3 and, in turn, ciliary disassembly [126]. VDAC3 is thought to be essential for UPS-mediated degradation of multiple targets during ciliary disassembly [127]. It has not been shown if TTK also regulates CDC20 in ciliary disassembly as it does during cell division.

### Overlap in microtubule regulators between mitosis and ciliary disassembly

In contrast to outer kinetochore proteins PLK1 and TTK, the KMN complex that binds the MTs in the kinetochores does not seem to be conserved in cilia. Instead, the ciliary tip module, positioned at the kinetochore corona, is thought to connect the ciliary membrane to the MTs. This docking is performed by the plus-end tracking tip proteins (+TIP), MT-binding proteins, and the IFT complex [128–130]. The plus-end tracking tip proteins MAPRE1 (EB1) and MAPRE3 (EB3) are conserved between kinetochores and cilia, but also play a role in the organization of other organelles [131–133]. At the kinetochores, they form a complex with CLASP1, CLASP2, CLIP1, CLIP2 and CKAP5 (also known as ch-TOG) [134, 135]. Even though these interacting proteins are not directly linked to ciliary disassembly, it is interesting to see that not CKAP5, but another TOG-domain protein, TOGARAM1, is involved in MT organization in cilia [136] and that recent studies have linked TOGARAM1 to Joubert syndrome [137]. Furthermore, CLASP1, CLASP2, CLIP1 and CLIP2 interactors are previously indicated to have a role in ciliary disassembly, including CENPE, CENPJ, PLK1, and dynein regulator PAFAH1B1. Together these data suggest that there might be a role for more of the +TIP proteins in ciliary disassembly, or that these cell cycle proteins have a counterpart with similar protein domain structures, which shadows their role in ciliary disassembly, as seen for CKAP5 and TOGARAM1.

In addition to the +TIP module, other MT regulators localized at the kinetochore corona during M phase have been indicated to position towards the cilia during G0/G1. These include, CENPE, CENPF, and the Dynein regulatory complex. Even though most of the family of CENP proteins are positioned at the inner kinetochore, CENPE and CENPF localize at the corona [138, 139]. CENPE is a plus-end directed motor protein that counteracts the minus-end directed Dynein proteins in positioning of the kinetochores and end-on attachment of the MTs [112, 140, 141]. CENPF is required for localization of the NDE1/NDEL1/PAFAH1B1 module at the kinetochores [111, 138]. On the other hand, during quiescence, CENPF localizes at the centrioles at the base of the cilia and has been shown to interact with CEP290 and ATF4 [118]. ATF4 is linked to skeletal and neuronal development [142, 143]. CEP290 is located at and just below the ciliary TZ during G0/G1 [144], and mutations in CEP290 cause a variety of ciliopathies (as discussed above). These examples underpin the importance of understanding the role of kinetochore proteins in ciliary function.

NDE1 regulates dynein and is required for the positioning and functioning of a variety of cell organelles. Dynein is a minus-end directed motor protein and together with dynactin important in MT elongation [145]. NDE1, together with NDEL1 and PAFAH1B1, regulates nuclear migration, Golgi localization, kinetochore positioning, and ciliary disassembly [146, 147]. The complex is recruited to the mother centriole by CENPJ [81]. Targeting of NDE1 or NDEL1 to the membrane through palmitoylation lowers the amount of cytoplasmic dynein and decreases dynein-mediated trafficking [146, 148]. Deletion of NDE1 results in a loss of membrane bound dynein, leading to microcephaly [149]. This same phenotype is seen with mutations in other proteins that regulate both the cell cycle and ciliary disassembly, among which CENPF, CENPJ, and KIF2A [86, 118, 150].

During G0/G1, NDE1 stimulates ciliary disassembly and cell cycle re-entry in an AURKA-dependent manner [147]. To allow ciliary elongation during ciliogenesis, CDK5 primes NDE1 for polyubiquitination by the E3 ligase FBXW7, targeting NDE1 for UPS-mediated degradation [151]. CDK5, NDE1 and PAFAH1B1 are essential in neural migration during brain development and mutations have been shown to cause lissencephaly [149, 152–155]. Of interest is that NDE1 and NDEL1 are located near the mother centriole at the base of the cilia during quiescence, while their downstream target PAFAH1B1, is located along the ciliary axoneme in a similar manner as Dynein [111, 146, 156] (Fig. 3b).

Dynein associates with the nuclear pore complex (NPC) members RANBP2 (NUP358), RANGAP1 and XPO1 (CARM1) at the kinetochores and this same association has been suggested to occur in the ciliary axoneme as a counter partner of the RAN/Importin complex that is required for nuclear and ciliary gating [107, 157–159]. This idea is supported by the fact that multiple nuclear pore (NUP) complex members have been identified to interact with IFTs at the base of the cilia in G0/G1 phase [19, 160]. For instance, NUP62 indirectly interacts with IQCB1 (NPHP5) and CEP290 (NPHP6) through NUP93, and it is targeted towards the ciliary tip in a KIF17-dependent manner [19]. Furthermore, inhibition of XPO1 has been shown to increase ciliary localization of SHH transcription factor GLI2 [160]. Lastly, it has been shown that RANBP2 is required for correct photoreceptor formation and functioning in mice [161]. It would be valuable to identify the entire Dynein/XPO1 complex in cilia, and to see if it functions in a similar manner as it does during the cell cycle.

A last interesting note is that most of the kinetochore corona cell cycle regulators are positioned at the base of the cilia during G0/G1, while many of the MT regulators are located along the ciliary axoneme or at the ciliary tip. Getting a better understanding of the localization and function of these modules in ciliary disassembly might prove valuable in the search for therapeutic targets.

### Overlapping mechanisms reveal cell cycle regulators with a potential role in ciliary resorption

Next to the MT regulators, there is a second group of proteins positioned at the kinetochore that plays a role in ciliary disassembly, being the APC/C and its regulators (Fig. 3a). Studying mitotic interactors of this complex in a ciliary-specific manner might reveal novel insight into the mechanism of ciliary resorption.

First, a role for BUB1B, ANAPC2 and CDC20 is well established in ciliary disassembly, however not all of the APC/C members and its regulators have been indicated to function in ciliary processes as of yet (Fig. 3b). For example, ANAPC2 and CDC20 have been shown to localize to the base of the cilia prior to and during ciliary disassembly, however, FZR1 localization at the base of the cilia could not be confirmed in quiescent cells or during ciliary disassembly [53]. To our knowledge, other APC/C components have not been studied with respect to ciliary localization thus far. Furthermore, the MCC is indicated to downregulate APC/C-CDC20 activity during G0/G1, and MCC-member BUB1B is a negative regulator of ciliary disassembly [54]. Nonetheless, since all MCC studies, thus, far focused on the cell cycle, it remains to be demonstrated the other MCC members, MAD2L1 and BUB3, are indeed also inhibiting CDC20 at the base of the cilia. The same accounts for CDC20-regulators BUB1 and the RZZ. During mitosis, BUB1 is required for BUB1B and RZZ recruitment to the kinetochores [114]. Here, the RZZ complex stimulates the MCC in downregulation of APC/C-CDC20 activity. Next to the MCC, the BUB1–PLK1 complex inhibits CDC20 in an MCC-independent manner, but it is unclear if this mechanism also functions during disassembly [162]. It would be interesting to see if MAD2L, BUB3, BUB1 and the RZZ have a similar mechanism of regulating BUB1B and CDC20 in ciliary disassembly by determining the exact spatiotemporal positions of these interactors. Second, another interesting target to study regarding ciliary resorption is BORA, since AURKA activation by BORA has been shown to be essential in mitosis, but it remains unclear if the same applies to ciliary resorption [163]. During anaphase, PLK1 marks BORA and AURKA for proteasomal degradation. The latter is regulated through the anaphase promoting complex/cyclosome (APC/C), which can polyubiquitinate AURKA after the complex is activated through FZR1. Another method through which the APC/C might influence AURKA in ciliary resorption is the APC/C E2 ubiquitin-conjugating enzymes UBE2C. This protein is required for polyubiquitination in the process of APC/C-dependent UPS-mediated degradation [164]. Inhibition of UBE2C leads to a reduction of AURKA phosphorylation. It remains unclear if it functions as an E2 after APC/C activation through CDC20 or FZR1 and if this process also occurs during ciliary resorption. Lastly, the later also applies to PTP4A3, which promotes AURKA degradation through APC/C-FZR1 in colorectal cancer progression [165]. Studying the role of BORA, UBE2C and PTP4A3 in a ciliary resorption-specific manner might reveal new cilium disassembly mechanisms that overlap with known mitotic mechanisms.

## Finding therapeutic targets by scrutinizing the conserved mechanisms

Looking at the type of disorders associated with defects in ciliary disassembly, we have to distinguish two groups of patients. On the one hand, the patients suffering from ciliopathies and developmental disorders, which are more often related to ciliogenesis and ciliary excision. These ciliopathies are caused by genetic predispositions leading to disruptions in embryonic development. Consequently, these are often embryonically lethal, hence, for this group of patients, finding the causative mutation is more relevant for genetic counselling of the expecting parents, than finding a therapeutic target. On the other hand, we have the group of patients showing MVA and different types of tumours. Although ciliogenesis defects are not typically linked to increased tumour formation [166], the opposite is true for ciliary disassembly proteins regulators [9, 167, 168]. The latter might be explained by the double role of ciliary resorption proteins in mitosis.

To get a better understanding of the possible therapeutic targets, the proteins involved in post-translational modifications (PTMs) might reveal interesting targets. PTMs are essential for proper ciliary disassembly. One example is microtubule acetylation, required for dimerization and auto-assembly to form the ciliary axoneme and, in turn, tubulin deacetylation by HDAC6 is required for ciliary disassembly [35, 169]. Another example is the highly diverse PLK1 that has opposing roles in both ciliary disassembly and mitosis based on its spatiotemporal position. Lastly, ubiquitination has been shown to increase upon the initiation of ciliary disassembly. Moreover, the relevance of UPS-mediated degradation in cell division and signal transduction has been known for almost 30 years now [170–173].

Getting a better understanding of the conservation between these two mechanisms is essential in the development of cancer treatment. One excellent example of this is the targeting of NEDD9 as an AURKA stabilizer in ciliary disassembly to potentiate AURKA treatment in breast tumours [37].

## Conclusion

In summary, many proteins involved in cilium disassembly also play a role in cell division. This conservation is not only seen for centrosomal proteins, but also for a range of other protein, among which kinetochore proteins and MT regulators. Misregulation of these proteins or mutations in the genes encoding them can lead to a variety of diseases affecting neuronal development, or can lead to tumour development across a range of different cancers. Getting a better understanding of the conservation between mitosis and cilium resorption might prove pivotal in developing therapeutic targets for these diseases.

## Supplementary Information

Below is the link to the electronic supplementary material.

Supplementary file1 (PDF 227 KB)

## Abbreviations

+TIP: Plus-end tracking tip protein complex
APC/C: Anaphase promoting complex/cyclosome
IFTA: Intraflagellar transport, retrograde
IFTB: Intraflagellar transport, anterograde
K-fibres: Kinetochore fibres
KMN: KNL1/MIS12/NDC80 complex
MCC: Mitotic checkpoint complex
MT: Microtubule
MTOC: Microtubule-organizing centre
NPC: Nuclear pore complex
NUP: Nuclear pore
PCM: Pericentriolar matrix
PTM: Post-translational modification
RZZ: KNTC1 (also known as ROD)-ZWILCH-ZW10 complex
SHH: Sonic hedgehog signalling pathway
TZ: (Ciliary) transition zone
UPS: Ubiquitin–proteasome system
WNT: Wingless and Int-1 signalling pathway
