# Supplementary material for: Moonlighting of mitotic regulators in cilium disassembly
Source: Cell Mol Life Sci. 2021 Apr 15;78(11):4955–72. doi: 10.1007/s00018-021-03827-5 (PMC8233288; doi:10.1007/s00018-021-03827-5)
Supplement: Supplementary file 1 — Supplementary file1 (PDF 227 KB) [file 18_2021_3827_MOESM1_ESM.pdf]

Electronic Supplementary Material for:

Moonlighting of mitotic regulators in cilium disassembly

Authors

Cenna Doornbos[1,2] and Ronald Roepman[1,2]

Affiliations

1 Department of Human Genetics, Radboud University Medical Center, Nijmegen, The Netherlands

2 Radboud Institute for Molecular Life Sciences, Radboud University Medical Center, Nijmegen, The Netherlands

Correspondence

Ronald Roepman, Department of Human Genetics, Radboud University Medical Center, Nijmegen, The Netherlands.

Email: Ronald.Roepman@radboudumc.nl

ORCID IDs

Cenna Doornbos: 0000-0002-0613-3651

Ronald Roepman: 0000-0002-5178-8163

Electronic Supplementary Material – Table 1 – Protein names, alternative names and Uniprot ACs

Name	Full name	Alternative name(s)	Abbreviation from alternative name	Uniprot AC
**ANAPC2**	Anaphase-promoting complex subunit 2	**APC2**, KIAA1406	-	Q9UJX6
**ANKS6**	Ankyrin repeat and SAM domain-containing protein 6	ANKRD14, PKDR1, SAMD6	-	Q68DC2
**ATF4**	Cyclic AMP-dependent transcription factor ATF-4	CREB2, TXREB, TaxREB67	Activating transcription factor 4	P18848
**AURKA**	Aurora kinase A	AIK, AIRK1, ARK1, AURA, AYK1, BTAK, IAK1, STK15, STK6	-	O14965
**BORA**	Protein aurora borealis	C13orf34, FLJ22624	-	Q6PGQ7
**BUB1**	Mitotic checkpoint serine/threonine-protein kinase BUB1	BUB1L, BUB1A	Budding uninhibited by benzimidazoles 1 homologue	O43683
**BUB1B**	Mitotic checkpoint serine/threonine-protein kinase BUB1 beta	**BUBR1**, MAD3L, SSK1	Budding uninhibited by benzimidazoles 1 homologue B	O60566
**BUB3**	Mitotic checkpoint protein BUB3	BUB3L	Budding uninhibited by benzimidazoles 3 homologue	O43684
**CALM1**	Calmodulin-1	CALM, CAM, CAM1	-	P0DP23
**CCP110**	Centriolar coiled-coil protein of 110 kDa	**CEP110**, CP110, KIAA0419	-	O43303
**CDC14A**	Dual specificity protein phosphatase CDC14A	**CDC14**, CDC14A1, Cdc14A2, DFNB105	Cell division cycle 14 homolog A	Q9UNH5
**CDC20**	Cell division cycle protein 20 homolog	**p55**, p55CDC, CDC20A	-	Q12834
**CDK5**	Cyclin-dependent-like kinase 5	CDKN5, PSSALRE	-	Q00535
**CDKN1A**	Cyclin-dependent kinase inhibitor 1	**p21**, CAP20, CDKN1, CIP1, MDA6, PIC1, SDI1, WAF1	-	P38936
**CENPE**	Centromere-associated protein E	KIF10, PPP1R61	-	Q02224
**CENPF**	Centromere protein F	**Mitosin**, HCP-1	-	P49454
**CENPJ**	Centromere protein J	**CPAP**, **SAS4**, LAP, LIP1	-	Q9HC77
**CEP104**	Centrosomal protein of 104 kDa	KIAA0562, JBTS25, GlyBP, RP1-286D6.4, CFAP256, ROC22	-	O60308
**CEP131**	Centrosomal protein of 131 kDa	**AZI1**, KIAA1118	-	Q9UPN4
**CEP290**	Centrosomal protein of 290 kDa	**NPHP6**, MKS4, BBS14, KIAA0373	-	O15078

Name	Full name	Alternative name(s)	Abbreviation from alternative name	Uniprot AC
**CEP41**	Centrosomal protein of 41 kDa	TSGA14, DKFZp762H1311, FLJ22445, JBTS15	-	Q9BYV8
**CKAP5**	Cytoskeleton-associated protein 5	**ch-TOG**, KIAA0097, TOG, TOGp	-	Q14008
**CLASP1**	CLIP-associating protein 1	KIAA0622, MAST1, hOrbit1	-	Q7Z460
**CLASP2**	CLIP-associating protein 2	KIAA0627, hOrbit2	-	O75122
**CLIP1**	CAP-Gly domain-containing linker protein 1	**CLIP170**, CYLN1, RSN	-	P30622
**CLIP2**	CAP-Gly domain-containing linker protein 2	**CLIP115**, CYLN2, KIAA0291, WBSCR3, WBSCR4, WSCR4	-	Q9UDT6
**DVL2**	Segment polarity protein dishevelled homolog DVL-2	DSH	-	O14641
**ESPL1**	Separin	ESP1, KIAA0165	Extra spindle poles-like 1 protein	Q14674
**FBXW7**	F-box/WD repeat-containing protein 7	FBW7, FBX30, SEL10, hCdc4, hAgo	-	Q969H0
**FZD1**	Frizzled-1	**FZ1**, FZE1, DKFZp564G072	-	Q9UP38
**FZR1**	Fizzy-related protein homolog	**CDH1**, FYR, FZR, KIAA1242	-	Q9UM11
**GLI2**	Zinc finger protein GLI2	THP, THP1, THP2, HPE9	-	P10070
**HDAC6**	Histone deacetylase 6	JM21, HD6, KIAA0901	-	Q9UBN7
**HIF1A**	Hypoxia-inducible factor 1-alpha	BHLHE78, MOP1, PASD8	-	Q16665
**INVS**	Inversin	**INV**, **NPHP2**	-	Q9Y283
**IQCB1**	IQ calmodulin-binding motif-containing protein 1	**NPHP5**, KIAA0036, OK/SW-cl.85	-	Q15051
**KIF17**	Kinesin-like protein KIF17	KIAA1405, KIF3X	-	Q9P2E2
**KIF24**	Kinesin-like protein KIF24	C9orf48, bA571F15.4, FLJ10933, FLJ43884	Kinesin superfamily protein 24	Q5T7B8
**KIF2A**	Kinesin-like protein KIF2A	KIF2, KNS2	-	O00139
**KNL1**	Kinetochore scaffold 1	**Blinkin**, **CASC5**, KIAA1570, D40/AF15q14, AF15q14, CT29	Kinetochore-null protein 1	Q8NG31
**KNTC1**	Kinetochore-associated protein 1	**ROD**, hROD, hsROD, KIAA0166	-	P50748
**MAD2L1**	Mitotic spindle assembly checkpoint protein MAD2A	MAD2, HSMAD2	-	Q13257
**MAPK14**	Mitogen-activated protein kinase 14	**p38α**, CSBP, CSBP1, CSBP2, CSPB1, MXI2, SAPK2A	-	Q16539
**MAPRE1**	Microtubule-associated protein RP/EB family member 1	**EB1**	-	Q15691

Name	Full name	Alternative name(s)	Abbreviation from alternative name	Uniprot AC
**MAPRE3**	Microtubule-associated protein RP/EB family member 3	**EB3**, RP3	-	Q9UPY8
**MIS12**	Protein MIS12 homolog	**MTW1**, MGC2488, KNTC2AP	-	Q9H081
**MTOR**	Serine/threonine-protein kinase mTOR	**FRAP**, FRAP1, FRAP2, RAFT1, RAPT1	Mammalian target of rapamycin	P42345
**NDC80**	Kinetochore protein NDC80 homolog	**HEC1**, HEC, KNTC2, TID3	Nuclear division cycle	O14777
**NDE1**	Nuclear distribution protein nudE homolog 1	NUDE, NUDE1	-	Q9NXR1
**NDEL1**	Nuclear distribution protein nudE-like 1	EOPA, MITAP1, NUDEL	-	Q9GZM8
**NEDD9**	Enhancer of filamentation 1	**HEF1**, CASL, CASS2, p105, NY-REN-12, HEF1-p55	Neural precursor cell expressed developmentally down-regulated protein 9	Q14511
**NEK1**	Serine/threonine-protein kinase Nek1	NY-REN-55, KIAA1901	-	Q96PY6
**NEK2**	Serine/threonine-protein kinase Nek2	NEK2A, NLK1, RP67, PPP1R111	-	P51955
**NEK8**	Serine/threonine-protein kinase Nek8	JCK, NEK12A	-	Q86SG6
**NPHP3**	Nephrocystin-3	KIAA2000	-	Q7Z494
**NPHP4**	Nephrocystin-4	POC10, KIAA0673	-	O75161
**NUP93**	Nuclear pore complex protein Nup93	KIAA0095	-	Q8N1F7
**OFD1**	Oral-facial-digital syndrome 1 protein	CXorf5, 71-7A, JBTS10	-	O75665
**PAFAH1B1**	Platelet-activating factor acetylhydrolase IB subunit alpha	**LIS1**, LIS, PAFAHA, MDCR, MDS	-	P43034
**PCM1**	Pericentriolar material 1 protein	PTC4	-	Q15154
**PIFO**	Protein pitchfork	C1orf88, FLJ23853	-	Q8TCI5
**PLK1**	Serine/threonine-protein kinase PLK1	PLK	-	P53350
**PTP4A3**	Protein tyrosine phosphatase type IV A 3	**PRL-3**, PRL3, PRLR	-	O75365
**PTTG1**	Securin	EAP1, PTTG, TUTR1	Pituitary tumor-transforming gene 1 protein	O95997
**RAB8A**	Ras-related protein Rab-8A	MEL, RAB8	-	P61006
**RAN**	GTP-binding nuclear protein Ran	ARA24, OK/SW-cl.81	-	P62826
**RANBP2**	E3 SUMO-protein ligase RanBP2	**NUP358**, ADANE	-	P49792
**RANGAP1**	Ran GTPase-activating protein 1	KIAA1835, SD, FUG1	-	P46060
**RPGRIP1L**	Protein fantom	**NPHP8**, MKS5, FTM, KIAA1005	-	Q68CZ1
**TMEM107**	Transmembrane protein 107	DC20, UNQ638/PRO1268	-	Q6UX40

Name	Full name	Alternative name(s)	Abbreviation from alternative name	Uniprot AC
**TMEM216**	Transmembrane protein 216	**MKS2**, HSPC244, JBTS2	-	Q9P0N5
**TOGARAM1**	TOG array regulator of axonemal microtubules protein 1	**Creserin**, Creserin-1, TOG2, FAM179B, KIAA0423	-	Q9Y4F4
**TP53**	Cellular tumor antigen p53	**p53**, LFS1	-	P04637
**TTK**	Dual specificity protein kinase TTK	**MPS1**, MPS1L, ESK, NCP, PYT, CT96, MPH1	Tyrosine/threonine-protein kinase	P33981
**UBE2C**	Ubiquitin-conjugating enzyme E2 C	**UBCH10**	-	O00762
**VDAC3**	Voltage-dependent anion-selective channel protein 3	HD-VDAC3	-	Q9Y277
**WNT5A**	Protein Wnt-5a	**WNT-5A**, hWNT5A	-	P41221
**XPO1**	Exportin-1	**CARM1**, CAF2, SPAC1805.17, SPAC1B2.01	-	O14980
**ZW10**	Centromere/kinetochore protein zw10 homolog	KNTC1AP	Zeste-White 10	O43264
**ZWILCH**	Protein zwilch	FLJ10036, KNTC1AP	-	Q9H900

Relevant protein information including protein name and Uniport AC for all recalled proteins. Alternative names in **bold** are often used interchangeably with the official protein name or these proteins have (recently) been renamed.
